# Supplementary material for: Complexome profiling of the Chlamydomonas psb28 mutant reveals TEF5 as an early PSII assembly factor
Source: Plant Cell. 2025 Mar 17;37(6):koaf055. doi: 10.1093/plcell/koaf055 (PMC12164590; doi:10.1093/plcell/koaf055)
Supplement: koaf055_Supplementary_Data [file koaf055_supplementary_data.zip › psb28_interactive.zip!/plots/intensity/qualityPlots/peptides/RAB5_Cre12.g517400.t1.2.html]

### Cre12.g517400.t1.2

Trivial name: RAB5  
SeqCoverage [median]: 27.70  
Mass: 22.12 kDa  
  
 

| Sequence | MissedCleavage | Mass [Da] | wtIntensity | muIntensity | Score |
| --- | --- | --- | --- | --- | --- |
| AAPLAELAQQQTAK | 0 | 1438.78 | 13866.89 | 4276.78 | 185.18 |
| ADLEGQR | 0 | 787.38 | 1449.38 | 787.10 | 114.78 |
| LVLLGEMGSGK | 0 | 1102.61 | 0.00 | 2486.58 | 67.99 |
| TSANVNELFEEIAR | 0 | 1591.78 | 515.36 | 0.00 | 173.19 |
| YHSLAPMYYR | 0 | 1299.61 | 982.86 | 902.88 | 52.73 |
|  |  |  | 16814.5 | 8453.3 |  |
